# Characteristics of delayed intracerebral hemorrhage after ventriculoperitoneal shunt insertion

**DOI:** 10.18632/oncotarget.17444

**Published:** 2017-04-27

**Authors:** Weiyi Gong, Li Xu, Peng Yang, Zhengquan Yu, Zhong Wang, Gang Chen, Shiming Zhang, Jiang Wu

**Affiliations:** ^1^ Department of Neurosurgery, The First People's Hospital of Kunshan, Suzhou, Jiangsu Province, 215300, China; ^2^ Department of Intensive Care Unit, The First Affiliated Hospital of Soochow University, Suzhou, Jiangsu Province, 215006, China; ^3^ Department of Emergency, The First Affiliated Hospital of Soochow University, Suzhou, Jiangsu Province, 215006, China; ^4^ Department of Neurosurgery, The First Affiliated Hospital of Soochow University, Suzhou, Jiangsu Province, 215006, China

**Keywords:** delayed intracerebral hemorrhage, hydrocephalus, ventriculoperitoneal shunt

## Abstract

**Background:**

Delayed intracerebral hemorrhage after ventriculoperitoneal (VP) shunt insertion is rare and has not been well investigated previously. Its characteristics is still unknown.

**Objective:**

We reported 12 patients with delayed intracerebral hemorrhage after VP shunt to investigate the potential risk factors and the outcome.

**Results:**

12 patients (1.59%) of all the 754 hydrocephalus had delayed intracerebral hemorrhage after VP shunt insertion. 4 patients were women and 8 patients were men, ranging in age from 50 to 76 years. The delayed cerebral hemorrhage from day 3 to day 7 post operation was diagnosed by repeated CT. The delayed intracerebral hemorrhage was significantly related to age, prior craniotomy operation history and manipulation of valve system (3–7 days). Neither gender sexuality nor potential risk factors for postoperative hemorrhage (including anticoagulation/antiplatelet status, liver disease, diabetes, hypertension), time of shunt attempt affected the happen of delayed intracerebral hemorrhage.

**Materials and Methods:**

The clinical characteristics including sex, age, anticoagulation/antiplatelet status, liver disease, diabetes, hypertension, craniotomy operation history, manipulation of valve system and time of shunt attempt of 754 patients who were surgically treated of VP shunt at the first affiliated hospital of Soochow University between 2007 and 2013 were reviewed retrospectively. The potential risk factors of the delayed intracerebral hemorrhage were statistically analyzed.

**Conclusions:**

This study summarizes the presentation and outcome of a series of 12 patients with delayed intracerebral hemorrhage after VP shunt. Age ≥ 60 years, prior craniotomy operation and manipulation of the valve system are statistically significant to the delayed hematoma secondary to VP shunt.

## INTRODUCTION

Cerebrospinal fluid (CSF) shunt procedures have dramatically reduced the morbidity and mortality of hydrocephalus, so the ventriculoperitoneal (VP) shunt is routinely used for CSF diversion. Postoperative complications after VP shunt placement include shunt obstruction, infection, catheter migration, subdural hematoma, seizures, and shunt malfunction [1, 2]. Intracerebral hemorrhage in the ventricle or in the parenchyma along the catheter path within 24 hours after the operation is an uncommon complication. The delayed hemorrhage which did not happen within 24 hours while occurred after 72 hours is rarer, there are only 7 cases been reported up to date [1, 3, 4]. However, the mortality of delayed intracerebral hemorrhage is high, ranging from 50% to 100% [3, 5]. Therefore, to improve clinical outcome of patients with delayed intracerebral hemorrhage after VP shunt, it may be important to understand the clinical features of the delayed intracerebral hemorrhage itself. In this study, we present clinical characteristics of the 12 patients with delayed intracerebral hemorrhage after VP shunt. Our data set has a uniquely large number of patients with delayed intracerebral hemorrhage, which enabled us to study the potential clinical risk factors and analyze the possible mechanisms.

## RESULTS

Table 1 shows the characteristics of 754 patients who received a ventriculoperineal shunt between 2007 and 2013, including sex, age, anticoagulation/antiplatelet status (Aspirin or Coumadin or Polivy), liver disease, diabetes, hypertension, craniotomy operation history, manipulation of valve system, and time of shunt attempt.

**Table 1:** Characteristics of 754 patients who received ventriculoperineal shunts between 2007 and 2013

	No. (%)
**Sex**	
Male	400 (53.1)
Female	354 (46.9)
**Age**	
≥ 60 Y	263 (34.9)
< 60 Y	491 (65.1)
**Anticoagulation/Antiplatelet status** (Aspirin or Coumadin or Polivy)	163 (22.0)
**Liver disease**	54 (7.2)
** Diabetes**	308 (40.8)
** Hypertension**	326 (43.2)
**Craniotomy operation history**	
Yes	79 (10.5)
No	675 (89.5)
**Manipulation of valve system(3-7day)**	
Yes	61 (8.1)
No	693 (91.9)
**Time of shunt attempt**	
> 1	154 (20.4)
= 1	600 (79.6)

We found 12 patients (1.59%) of all the 754 hydrocephalus had delayed intracerebral hemorrhage after ventriculoperitoneal shunt insertion (summarized in Table 2). 4 patients were women and 8 patients were men, ranging in age from 50 to 76 years. 3 patients suddenly became unconscious, and 5 patients developed a headache and deterioration, and the other 4 patients were disclosed by routine follow-up CT. The delayed cerebral hemorrhage was diagnosed by CT from postoperative day 3 to day 7. The cerebral hemorrhage was all intraparenchymal and/or intraventricular hemorrhage. 3 patients (the patient 1 died before the second operation) were again operated for evacuation of the hemorrhage and the other 9 patients were treated conservatively. 5 patients showed favorable recovery at six months after the onset of hemorrhage, and 7 patients were unfavorable outcomes.

**Table 2:** Summary of 12 patients with delayed intracerebral hemorrhage after ventriculoperitoneal shunt

No.	Age	A/A status	Liver disease Diabetes Hypertension	Craniotomy operation history	Shunt operation site operative finding	manipulation of valve system (day)	Onset day	Treatment	Outcome (GOS)
1	62/M	no	yes	HICH	AH uneventful	no	3	Operation (undone)	1
2	64/F	no	no	trauma	PH uneventful	no	3	conservative	3
3	76/M	no	yes	SAH	AH uneventful	yes(5)	7	conservative	3
4	50/M	no	no	SAH	AH uneventful	no	3	operation	2
5	61/F	no	no	trauma	AH uneventful	no	4	conservative	4
6	67/M	no	no	meningitis	AH uneventful	yes(3)	5	conservative	3
7	65/M	no	no	no	AH uneventful	yes(5)	7	conservative	4
8	61/M	no	no	no	AH uneventful	yes(3)	4	conservative	5
9	60/M	no	no	trauma	AH uneventful	no	3	conservative	4
10	53/F	no	no	SAH	PH uneventful	no	4	operation	2
11	68/F	no	no	No	AH uneventful	no	5	conservative	5
12	61/M	no	yes	SAH	AH uneventful	no	5	operation	3

A/A: Anticoagulation/Antiplatelet, AH: anterior horn of lateral ventricle, PH: posterior horn of lateral ventricle, SAH: subarachnoid hemorrhage, HICH: hypertensive intracerebral hemorrhage.

Table 3 shows a clinical relationship between with delayed intracerebral hemorrhage and without delayed intracerebral hemorrhage of patients. The delayed intracerebral hemorrhage was significantly associated with age, craniotomy operation history and manipulation of valve system (3–7 days). Neither gender sexuality nor potential risk factors for postoperative hemorrhage (including anticoagulation/antiplatelet status, liver disease, diabetes, hypertension), time of shunt attempt affected the happen of delayed intracerebral hemorrhage.

**Table 3:** Clinical relationship between with delayed intracerebral hemorrhage and without intracerebral hemorrhage of 754 patients

	No. (%) without intracerebral hemorrhage (*n* = 742)	No. (%) with intracerebral hemorrhage (*n* = 12)	*P* value (test) (Fisher's exact test)
**Sex**			0.3947
Male	392(52.8)	8(66.7)	
Female	350(47.2)	4(33.3)	
**Age**			0.0008 *
≥ 60 Y	253(34.1)	10(83.3)	
< 60 Y	489(65.9)	2(16.7)	
**Anticoagulation/Antiplatelet status**			0.0795
Yes	163(22.0)	0(0)	
No	579(78.0)	12(100.0)	
**Liver disease**			0.5930
Yes	53(7.1)	1(8.3)	
No	689(92.9)	11(91.7)	
**Diabetes**			0.1360
Yes	306(41.2)	2(16.7)	
No	436(58.8)	10(83.3)	
**Hypertension**			0.2490
Yes	323(43.5)	3(25.0)	
No	419(56.5)	9(75.0)	
**Craniotomy operation history**			0.0001 *
Yes	70(9.4)	9(75.0)	
No	672(90.6)	3(25.0)	
**Handling of valve system(3–7 days)**			0.0117 *
Yes	57(7.7)	4(33.3)	
No	685(92.3)	8(66.7)	
**Time of shunt attempt**			0.1397
> 1	154(20.8)	0(0)	
= 1	588(79.2)	12(100.0)	

### Illustrative cases

#### Patient 1

**Figure 1:**
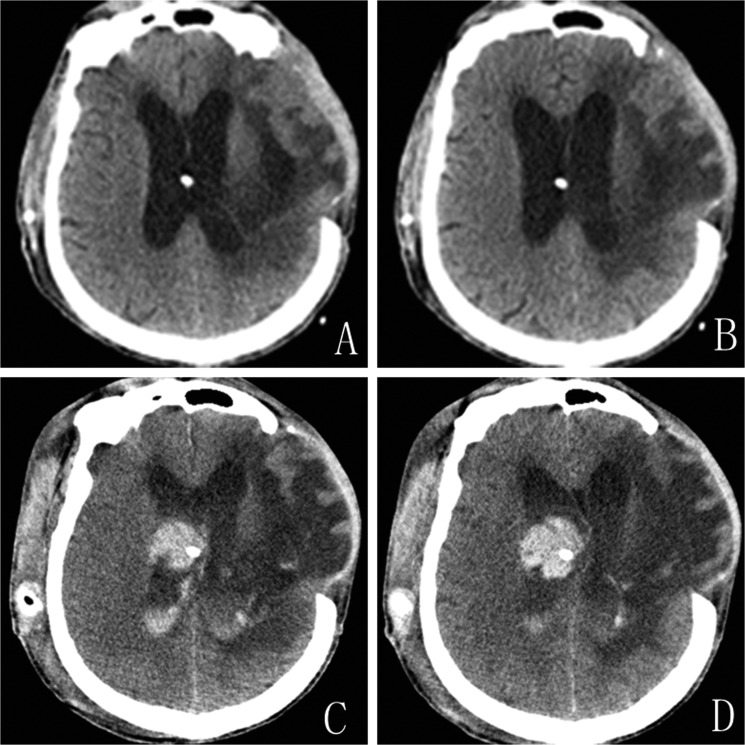
Patient 1. (**A** and **B**) CT scans obtained one day after shunt placement demonstrating shrinking ventricles and no hematoma. (**C** and **D**) CT on postoperative day three showed intraventricular hemorrhage along the path of ventricular catheter.

A 62-year-old male underwent a decompressive left frontotemporal craniectomy for hypertensive intracerebral hemorrhage 3 months previously. CT showed hydrocephalus with interstitial edema capping the frontal horns of the lateral ventricles. Preoperative neurological examination was unremarkable. A VP shunt was placed with a Medtronic Strata Programmable Valve System (pressure level, 1.5). A ventricular catheter was inserted into the anterior horn of the right lateral ventricle at the first attempt. 24 hours after surgery, CT imaging was normal and the pressure of the left decompressive window decreased (Figure 1A, 1B). On postoperative day 3, the pressure of decompressive window increased and the patient became unconscious. Emergent CT showed a large hematoma along the path of the catheter associated with an appreciable intraventricular hemorrhage (Figure 1C, 1D). Unfortunately, the patient died suddenly before the evacuation of the hematoma.

#### Patient 4

**Figure 2:**
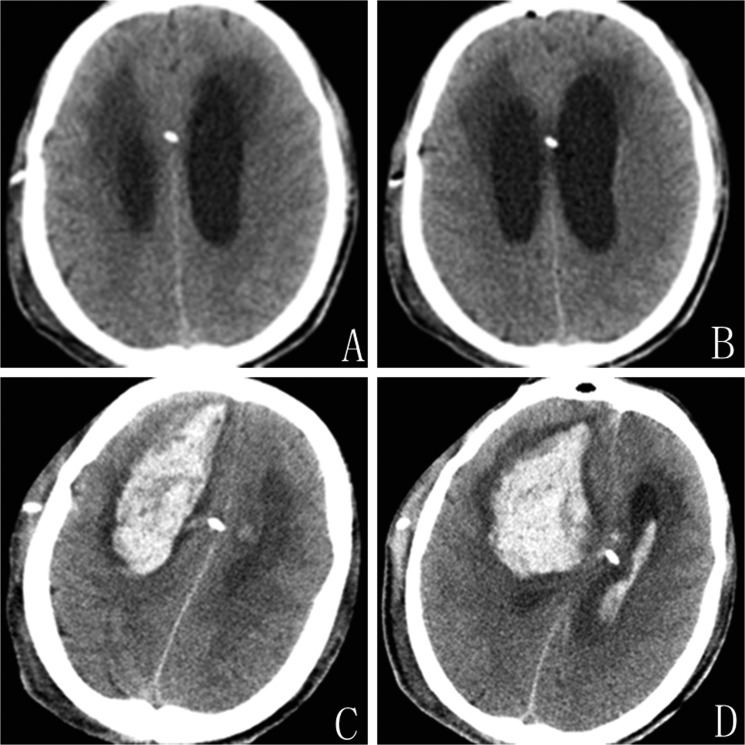
Patient 4. (**A** and **B**) CT showed no evidence of hemorrhage on postoperative day one. (**C** and **D**) CT on postoperative day four indicate large intraventricular and intracerebral hematoma along the path of ventricular catheter.

A 50-year-old male without previous diseases had suffered subarachnoid hemorrhage (SAH) due to rupture of a right vertebral artery aneurysm which was clipped. The subsequent hydrocephalus was treated with a VP shunt in the right anterior horn of the lateral ventricle. The operative procedure was uneventful. The patient recovered well and routine follow-up CT on postoperative day 1 showed no hematoma, but the catheter is near to the middle line (Figure 2A, 2B). On postoperative day 5, the patient suddenly developed a headache and seizure while urinating, and then became less alert soon. The new CT revealed a large hematoma around the ventricular catheter with opening to the ventricular system (Figure 2C, 2D). The clinical course was unfavorable and the patient was operated again for evacuation of the intraparenchymal and intraventricular hemorrhage. The subsequent improvement was slight. He remained unconscious, with open eyes, but without verbal response and submission.

#### Patient 7

**Figure 3:**
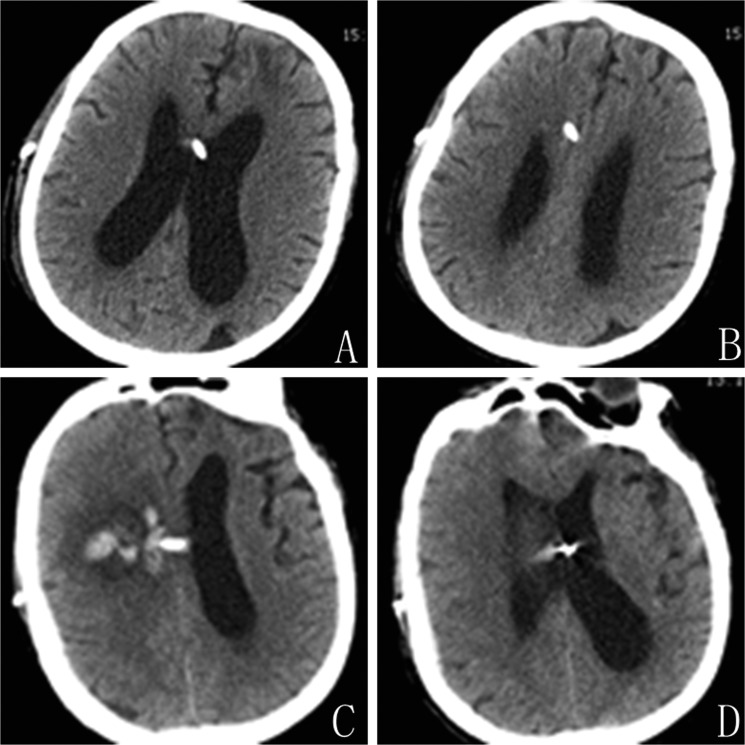
Patient 7. (**A** and **B**) Routine CT scans acquired three days after shunt showed shrinking ventricles. (**C** and **D**) CT on postoperative day seven showed hemorrhage along the path of ventricular catheter.

A 65-year-old male complained of unstable walking and reaction retardation for 3 months. CT showed hydrocephalus with moderately enlarged lateral ventricles. A VP shunt was placed via a right frontal horn puncture at the first attempt. Head CT performed shortly after the operation and three days later demonstrated normalization of the ventricular size without hematoma, we changed its opening pressure (1.5 to 1.0). The routine follow up CT acquired 7 days after the operation revealed a small hematoma along the catheter (Figure 3), but no neurological deficits. The patient was treated conservatively and recovered gradually.

## DISCUSSION

This study demonstrated a uniquely large number of 12 patients with the delayed hemorrhage after VP shunt which is related to age, craniotomy operation history and manipulation of the valve system. Furthermore, the mechanism of delayed hemorrhage may be caused by the erosion of cerebral vessels from the catheter, it is particularly prone to happen during collapse of the ventricle and/or with craniotomy operation history of the aged (≥ 60 years) patients.

The delayed intracerebral hemorrhage is an uncommon complication of VP shunt placement. Matsumura reported the first case in 1985 [4], and there are only 7 cases can be found in literatures [4–8]. In this article, we reported 12 patients with delayed intracerebral hemorrhage. In addition, the incidence in our series is 1.59% (12/754) which is higher than that given in previous reports [4–8]. All the patients in our study having CT scans 3–7 days after surgery might affect the prevalence of this rare complication. Our study also indicated a predominance of men (66.7%). The observations of this study are consistent with previous reports [8]. However, the predominance of sex has no statistical significance.

Previous studies suggested that mortality of the delayed intracerebral hemorrhage is high [3, 5, 8]. Zhou reported two cases of delayed hemorrhage after ventricular shunting procedures, the mortality is 50% [8]. In our patients, the mortality is only 8.3%, whereas 58.3% patients (7/12) were unfavorable which seems consistent with previous studies [3, 5]. Because of the high disability rate, it is important to know when the delayed intracerebral hemorrhage happens. The previous 6 cases (6/7, 86%) happened from postoperative day 3 to day 7 [4–8]. In all of our patients, the head CT performed 24 hours after surgery showed no hematoma, all the delayed hematoma in our series occurred within 7 days after surgery. We suggest that repeat imaging should be done during postoperative day 3 to day 7 routinely, emergency CT is necessary if the patient complains additional symptoms.

The potential risk factors of acute intracerebral hemorrhage secondary to VP shunt placement are coexistent bleeding disorder, shunt induced disseminated intravascular coagulation, disruption of an intracerebral tumor, disruption of intracerebral vessel, hemorrhage from an occult vascular malformation and head trauma occurring shortly after shunt placement [9–11]. However, these risk factors failed to explain the delayed hemorrhage. According to previous studies, some results suggested that the fragility of brain and the erosion of a cerebral blood vessel might be involved in the risk factors [7, 8]. The observations of this study indicated that age, craniotomy operation history and manipulation of the valve system might increase the risk of the delayed hemorrhage. (1) Age ≥ 60 years: We find that the age of more than 60 years significantly increase the risk of delayed intracerebral hemorrhage. This might be caused by the cerebral vessels of aged patients are easy to erosio. (2) Craniotomy operation history: 9 patients (75%) in our study had the history of craniotomy operation. The prior craniotomy operation may make the arachnoid adhesive, and the adhesive arachnoid with small cerebral vessels is prone to bleeding after the place of catheter. (3) Manipulation of the valve system: Handling of the valve system in early phase (3–7 days after operation) may be another risk factor. As we all know, ventricular size will reduce after catheter insertion, especially following the manipulation of the valve, the ventricle even collapsed. The collapse of ventricle might induce the contact between the ventricular catheter and cerebral blood vessel of parenchyma along the catheter path. Furthermore, when the ventricular size significantly reduced, the catheter even may erode the choroid plexus or the ventricular wall, especially when the place of the catheter is near to the midline or deep in the ventricle. This may explain the delayed hemorrhage which we encountered and the present cases reported. This study has limitations that it is a retrospective study and focused on limited samples of single medical center. Thus, in future prospective studies of multiple center, we believe that more cases of these ‘infrequent’ events will be reported with sufficient knowledge of their clinical characteristics.

## MATERIALS AND METHODS

We retrospectively reviewed a total of 754 patients whose hydrocephalus were treated surgically at the First Affiliated Hospital of Soochow University during the period from 2007 to 2013. Computed tomography (CT) showed markedly enlarged ventricles. There were no space-occupying lesions on magnetic resonance imaging (MRI). Lumbar puncture revealed transparent CSF before the operation. A diagnosis of hydrocephalus was made, blood coagulation status which including medication history (Aspirin or Coumadin or Polivy), liver disease, diabetes, hypertension was evaluated. After anticoagulation/antiplatelet drug was stopped for one week and the blood coagulation status was normal, a ventricular catheter was inserted into the lateral ventricle through a frontal or occipital burrhole after test tapping. The burrhole was located at the coronal suture at the mid-papillary line for frontal access to the ventricle or approximately 5 cm above the inion and 6 cm lateral from the midline for occipital access. Getting transparent CSF after one time of shunt attempt means uneventful operative finding. All the surgically treated patients had CT scans from the third postoperative day to the seventh day, if the size of ventricle was not improved significantly, the handling of valve system was done within 7 days after operation and the valve system was pressed 30 times every 8 hours. A total of 12 patients with delayed cerebral hemorrhage after placement among 754 surgery were disclosed by CT scan. The clinical outcome was categorized according to the Glasgow Outcome Scale (GOS). GOS scores were reported as follows: 5, good recovery; 4, moderate disability; 3, severe disability; 2, vegetative state; and 1, death. We assessed the outcome according to the scores as favorable (5 or 4) or unfavorable (3, 2 or 1). Statistical analysis was performed by use of Fisher's exact test, *P* < 0.05 was required for statistical significance. All computations were carried out by use of SPSS 19.0 statistical software.

## CONCLUSIONS

In summary, we reported 12 delayed hemorrhages secondary to VP shunt which is the largest study performed to date. The presence of delayed hematoma is from day 3 to day 7 after surgery. To our knowledge, this is the first study that addresses the risk factors for delayed post-shunting hemorrhage, including age ≥ 60 years, craniotomy operation history and manipulation of the valve system.
